# Function of high-mobility group A proteins in the DNA damage signaling for the induction of apoptosis

**DOI:** 10.1038/srep31714

**Published:** 2016-08-19

**Authors:** Ryosuke Fujikane, Kayoko Komori, Mutsuo Sekiguchi, Masumi Hidaka

**Affiliations:** 1 Department of Physiological Science and Molecular Biology, Fukuoka Dental College, Fukuoka, 814-0193, Japan; 2 Department of Molecular Biology, Biomolecular Engineering Research Institute, Suita, 565-0874, Japan; 3 Advanced Science Research Center, Fukuoka Dental College, Fukuoka, 814-0193, Japan

## Abstract

O^6^-Methylguanine produced in DNA can pair with thymine during DNA replication, thus leading to a G-to-A transition mutation. To prevent such outcomes, cells harboring O^6^-methylguanine-containing mispair undergo apoptosis that requires the function of mismatch repair (MMR) protein complex. To identify the genes involved in the induction of apoptosis, we performed gene-trap mutagenesis and isolated a clone of mouse cells exhibiting an increased resistance to the killing effect of an alkylating agent, *N*-methyl-*N*-nitrosourea (MNU). The mutant carries an insertion in the *Hmga2* gene, which belongs to a gene family encoding the high-mobility group A non-histone chromatin proteins. To elucidate the function of HMGA proteins in the apoptosis pathway, we introduced siRNAs for *HMGA1* and/or *HMGA2* into human HeLa MR cells defective in O^6^-methylguanine-DNA methyltransferase. *HMGA1*- and *HMGA2*-single knockdown cells showed an increased resistance to MNU, and *HMGA1*/*HMGA2*-double knockdown cells exhibited further increased tolerance compared to the control. The phosphorylation of ATR and CHK1, the appearance of a sub-G_1_ population, and caspase-9 activation were suppressed in the knockdown cells, although the formation of mismatch recognition complex was unaffected. These results suggest that HMGA family proteins function at the step following the damage recognition in the process of apoptosis triggered by O^6^-methylguanine.

The modification of DNA bases occurs spontaneously during normal cell growth, and the rate of formation of modified bases increases considerably when cells are exposed to certain chemical agents. O^6^-Methylguanine (O^6^-meG) is one such base produced by the action of simple alkylating agents, such as *N*-methyl-*N*-nitrosourea (MNU) and *N*-methyl-*N*′-nitro-*N*-nitorosoguanidine (MNNG), and is of particular importance in terms of mutagenicity and cytotoxicity. O^6^-MeG can pair with thymine as well as cytosine during DNA replication, inducing the G:C to A:T transition mutation^1^,^2^. To preserve the genome integrity, a wide range of organisms possesses a specific repair protein, O^6^-methylguanine-DNA methyltransferase (MGMT), which transfers a methyl group from O^6^-meG to a catalytic cysteine residue in the protein^3^,^4^,^5^. In addition to the repair reaction catalyzed by MGMT, the apoptosis of cells carrying the mutagenic O^6^-meG is another strategy for organisms to preserve genomic integrity^6^. O^6^-MeG:T mispair, produced during DNA replication, is specifically recognized by a mismatch repair (MMR) protein complex, composed of MutSα (a heterodimer of MSH2 and MSH6) and MutLα (a heterodimer of MLH1 and PMS2)^6^,^7^,^8^. The resulting MMR complex formed on the damaged chromatin is the mediator of apoptosis induction.

In cells treated with MNU, the activation of the DNA damage response, which involves the phosphorylation of checkpoint kinases, such as ATR, CHK1, ATM, and CHK2, have been shown to take place in an MMR protein-dependent manner^9^,^10^,^11^. After the checkpoint activation, depolarization of the mitochondrial membrane followed by the activation of caspase-9 and caspase-3 occurs in the pathway of O^6^-mG-induced apoptosis^12^. The MMR complex may activate checkpoint kinases and lead to apoptosis through direct interaction with ATR on O^6^-meG:T-containing DNA^13^. Alternatively, secondary DNA lesions, produced by a futile cycle of repair reaction by MMR proteins, might induce apoptosis^8^,^14^. In this regard, it is important to know which proteins function directly, after the MMR complex formation, in the induction of apoptosis.

Gene-trap mutagenesis is a useful technique for identifying genes that function in various cellular processes. By applying this method to a mouse-derived cell line, we previously isolated the new genes *Mapo1* and *Mapo2*, which are involved in the O^6^-meG-induced apoptosis^12^,^15^. The inhibition of the expression of these genes in human-derived cells suppressed the induction of several apoptosis-related activities, including the dimerization of Bak, mitochondrial membrane depolarization, and caspase-3 activation, although the activation of the DNA damage response was unaffected. Further investigations revealed that MAPO1 forms a complex with foliculin (a tumor suppressor protein encoded by *FLCN*) and AMP-activated protein kinase (AMPK; an energy sensor composed of AMPKα, β, and γ subunits), implying a role of MAPO1 in the signal transduction pathway in the cytoplasm^16^,^17^.

By extending the gene-trap mutagenesis screening, we found that high-mobility group A (HMGA) family proteins are involved in MNU-induced apoptosis. HMGA proteins are non-histone chromatin proteins encoded by two distinct genes: *HMGA1* and *HMGA2*^18^. HMGA proteins *per se* do not possess intrinsic transcriptional regulatory activity; however, by interacting with transcription factors they have ability to alter chromatin structure and regulate gene transcription^19^,^20^. HMGA family proteins were found to be over-expressed in different types of malignant tumors. Furthermore, the overexpression of HMGA was shown to suppress the DNA repair ability of the cells, thereby sensitizing cells to DNA damage^21^,^22^,^23^. HMGA2 overexpression is also involved in doxorubicin-induced G_2_-M cell cycle delay and induces persistent phosphorylation of H2AX by modulating the activation of ATM^24^. These data suggest possible roles of HMGA proteins in the signaling pathway in response to DNA damage.

In this report, we present evidence supporting a novel function of the HMGA family proteins HMGA1 and HMGA2 at an early step of the apoptosis pathway triggered by O^6^-meG.

## Results

### Isolation of a mouse cell line defective in *Hmga2*

**Figure 1:**
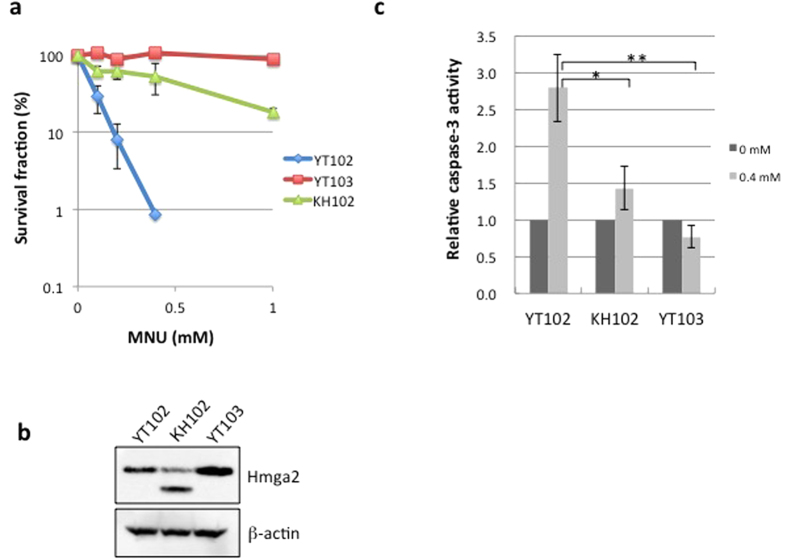
The phenotypic analyses of the mouse *Hmga2*-deficient cell line KH102. (**a**) The survival rates of three mouse cell lines after treatment with MNU. YT102 (*Mgmt*^−/−^, blue diamond), YT103 (*Mgmt*^−/−^ *Mlh1*^−/−^, orange square), and KH102 (*Mgmt*^−/−^ *Hmga2*^+/−^, green triangle) cell lines were treated with various concentrations of MNU. The numbers of colonies were counted, and the survival fractions were determined. The mean values obtained from at least three independent experiments and the standard deviations are shown. (**b**) The expression of Hmga2 protein in the three cell lines. The whole extracts were used to detect Hmga2 protein by immunoblotting with a specific antibody. (**c**) The activation of caspase-3 after MNU treatment. The caspase-3 activities were measured at 72 h after treatment with or without 0.4 mM MNU. The values from MNU-treated samples were divided by the values from the untreated samples, and the relative caspase-3 activities were plotted. The mean values obtained from three independent experiments and the standard deviations are shown with bars (dark gray for untreated control, light gray for cells treated with 0.4 mM MNU). Significance values of *P < 0.002 and **P < 0.001 relative to YT102.

Retrovirus-mediated gene-trap mutagenesis was performed to isolate cells defective in genes functioning in the MNU-induced apoptosis pathway. YT102 cells, established from lung fibroblast of *Mgmt*-knockout mice, were infected with the gene-trap vector containing a promoterless hygromycin B resistance gene, and hygromycin-resistant (Hyg^r^) cells were selected. From the collection of Hyg^r^-cells, MNU-resistant clones were isolated as candidates that have an insertional mutation of the vector sequence in genes involved in O^6^-meG-induced apoptosis. One of the isolated clones, termed KH102, exhibited significant resistance to MNU in comparison to the parental cell line YT102, although the level of resistance was lower than that of the YT103 cell line, which is defective in both *Mgmt* and *Mlh1* (Fig. 1a). To identify the gene disrupted in KH102, an inverse polymerase chain reaction (PCR) was performed, which amplifies DNA fragments spanning the junctions between the genomic DNA and the integrated vector sequences, and the nucleotide sequences of the amplified fragments were determined. A database search revealed that the vector DNA was integrated into a sequence corresponding to the large third intron between exons 3 and 4 of the gene encoding high-mobility group AT-hook 2 (HMGA2), located on mouse chromosome 10. An immunoblotting analysis revealed that the expression level of Hmga2 in KH102 was less than half that in YT102, and a band for a protein with a smaller molecular mass was detected incidentally (Fig. 1b), suggesting the expression of a truncated form of Hmga2 from a gene-trapped allele of the gene.

To clarify the involvement of HMGA2 in the induction of apoptosis triggered by O^6^-meG, we measured the caspase-3 activity in three cell lines after administration of 0.4 mM MNU. As shown in Fig. 1c, the caspase-3 activity in YT102 cells increased to more than 2.5 times the level of untreated cells, whereas no increase was observed in *Mlh1*-defective YT103 cells. In KH102 cells, the activation of caspase-3 was hardly detected, even after the treatment with MNU. These results suggest that *Hmga2*-deficient KH102 cells acquired resistance to MNU, due to the inability to induce apoptosis.

### Involvement of human HMGA proteins in O^6^-methylguanine-induced apoptosis

**Figure 2:**
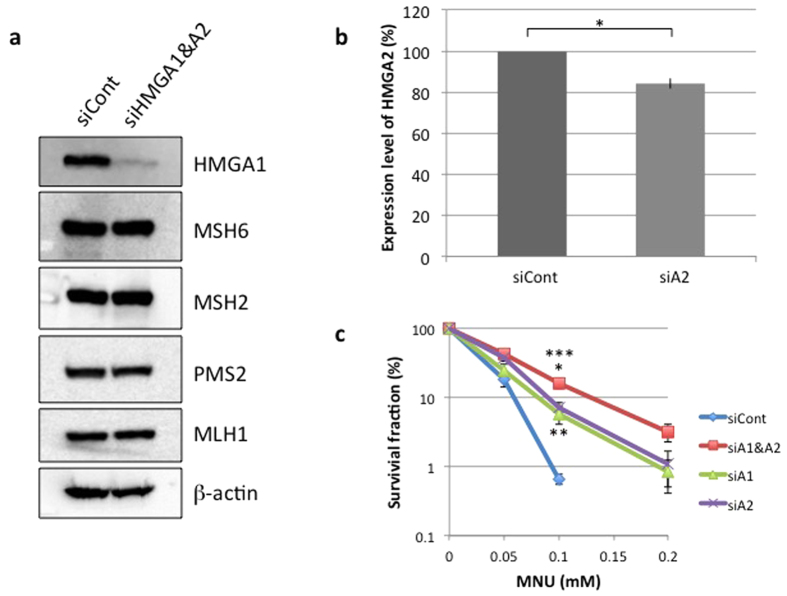
The analyses of HeLa MR cells transfected with siRNAs specific for HMGA family genes. (**a**) The expression of HMGA1 and mismatch repair (MMR) proteins in *HMGA*-knockdown cells. The whole-cell extracts were subjected to immunoblotting to detect HMGA1 and MMR proteins using specific antibodies. β-actin was a loading control. (**b**) The expression of *HMGA2* in HeLa MR and *HMGA2*-knockdown cells as measured by quantitative real-time PCR. Significance value of *P < 0.001 relative to siCont. (**c**) The survival rates of three types of *HMGA-*knockdown HeLa MR cells after MNU treatment. The mean values obtained from at least three independent experiments and the standard deviations are shown. Red square, *HMGA1*/*HMGA2*-double knockdown; green triangle, *HMGA1*-knockdown; purple cross, *HMGA2*-knockdown; blue diamond, negative control. Significance values of *P < 0.005 and **P < 0.05 relative to siCont, and ***P < 0.05 relative to siA1 and siA2.

The mammalian HMGA family is composed of two members—HMGA1 and HMGA2—and they have some redundant molecular functions^25^. To see if either or both proteins function in the O^6^-meG-induced apoptosis, siRNAs specific for *HMGA1* and/or *HMGA2* were introduced into HeLa MR cells. The expression of *HMGA1* was significantly reduced in siHMGA1/siHMGA2-double knockdown cells compared to that in cells treated with siCont (Fig. 2a). A quantitative real-time PCR analysis revealed a 16% reduction in the *HMGA2* mRNA level relative to that of the control in siHMGA2-transfected cells (Fig. 2b). It should be noted that, under these conditions, the expression of the MMR proteins MSH2, MSH6, MLH1, and PMS2 was unaffected (Fig. 2a). siRNA-treated cells were exposed to various doses of MNU, and the survival fractions were determined. As shown in Fig. 2c, *HMGA1*- or *HMGA2*-single knockdown cells showed considerable degrees of resistance to the treatment with MNU. Furthermore, *HMGA1*/*HMGA2*-double knockdown cells exhibited further increase in the resistance level to MNU. These results suggest that both HMGA proteins may be involved in the process of apoptosis triggered by O^6^-meG.

### Formation of mismatch recognition complex in *HMGA*-knockdown cells

**Figure 3:**
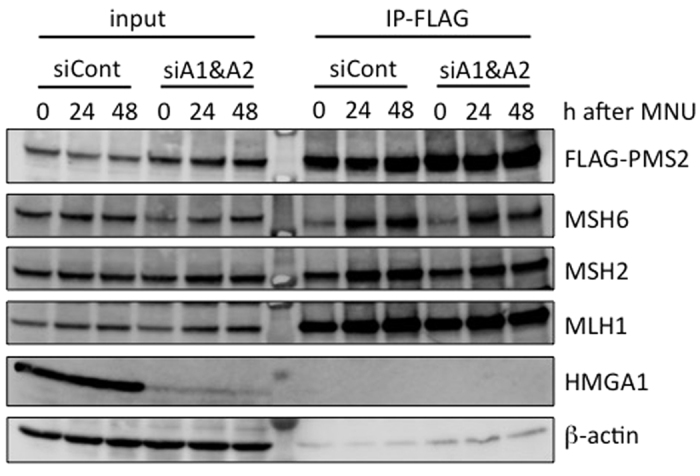
The formation of an MMR complex in *HMGA*-knockdown cells after MNU treatment. HeLa MR cells expressing Flag-tagged PMS2 were treated with control- or *HMGA*-siRNA and then exposed to 0.2 mM MNU for 1 h. The chromatin extracts (input) were prepared from cells collected at 0, 24, and 48 h after MNU treatment and applied to immunoprecipitation with anti-Flag M2 antibody beads (IP-FLAG). The materials were subjected to SDS–PAGE, transferred to a membrane, and immunoblotted using specific antibodies.

An MMR complex composed of MutSα (MSH2 and MSH6) and MutLα (MLH1 and PMS2) is formed on chromatin when human cells are exposed to MNU^7^. To see if such a complex formed in *HMGA*-knockdown cells, we performed immunoprecipitation experiments using chromatin extracts prepared from HeLa MR cells stably expressing Flag-tagged PMS2. As shown in Fig. 3, using an anti-Flag antibody, the stable association of MLH1 and MNU-induced interactions of MSH2 and MSH6 with Flag-PMS2 were detected in siCont-transfected cells. Even when both siHMGA1 and siHMGA2 were introduced, the same level of complex formation was achieved. It appears that HMGA proteins are dispensable for the formation of mismatch recognition complex. We therefore next examined if *HMGA*-knockdown cells have any defects in downstream events following mismatch recognition.

### Suppression of DNA damage response in *HMGA*-knockdown cells

**Figure 4:**
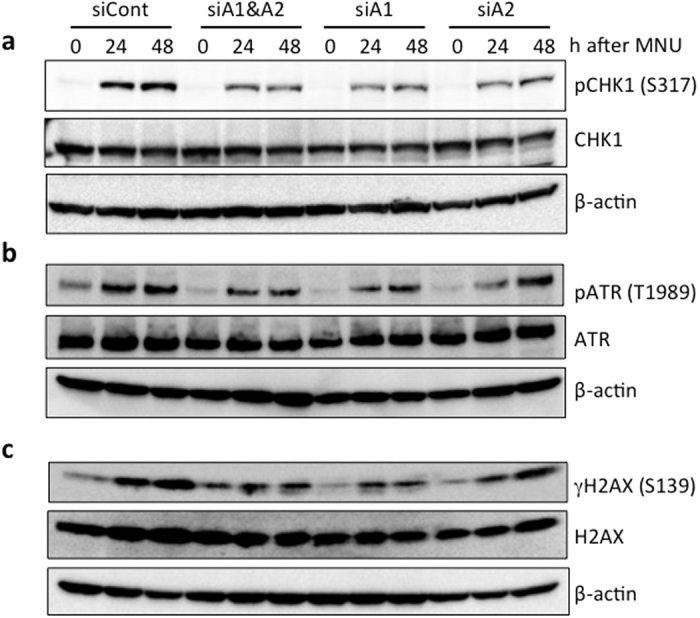
The effects of *HMGA*-knockdown on the activation of CHK1. Three types of *HMGA*-knockdown cells were treated with 0.4 mM MNU for 1 h and then collected at the indicated times. The whole-cell extracts were prepared and subjected to SDS-PAGE, followed by immunoblotting using the specific antibodies indicated. (**a**) Detection of serine 317-phosphorylated and total CHK1 protein. (**b**) Detection of threonine 1989-phosphorylated and total ATR. (**c**) Detection of serine 139-phosphorylated (γH2AX) and total H2AX. β-actin was the loading control.

DNA damage responses, including the phosphorylation of checkpoint kinases, such as ATR and CHK1, occur in the course of MNU-induced apoptosis^26^. The activation of those kinases is dependent on O^6^-meG among various types of alkylated bases, as the phosphorylation of CHK1 in the HeLa S3 cell line, which is proficient in MGMT, is not observed without adding O^6^-benzylguanine, a specific inhibitor of MGMT, even after treatment with MNU (Supplementary Fig. S1). To determine if defects in HMGA proteins affect these processes, the phosphorylation status of CHK1 in *HMGA*-knockdown cells was analyzed. HeLa MR cells, transfected with control siRNA (siCont) or *HMGA*-specific siRNAs, were treated with 0.4 mM MNU for 1 h, followed by cultivation for 24 and 48 h, and the whole-cell extracts were subjected to an immunoblotting analysis (Fig. 4a). In the siCont-transfected cells, the phosphorylation of the serine residue at position 317 of CHK1 was clearly detected after MNU treatment. When a mixture of siHMGA1 and siHMGA2 (siA1&A2) was introduced, the levels of the phosphorylated form of CHK1 were reduced at both 24 and 48 h after the drug administration. Almost the same levels of reduction were achieved in *HMGA1* (siA1)- and *HMGA2* (siA2)-single knockdown cells. It should be noted that the total amounts of CHK1 were unaffected, even after these treatments. Similar results were obtained in the case of ATR, which is a master kinase of ATR/CHK1-mediated DNA damage response. In comparison with the control (siCont), the phosphorylation of the threonine residue at position 1989 of ATR after treatment of MNU was suppressed when siRNAs targeting either or both *HMGA* genes were introduced (Fig. 4b). Furthermore, the phosphorylation of histone H2AX, a downstream event of the ATR/CHK1 axis, was attenuated in the *HMGA*-knockdown cells (Fig. 4c). In the mouse KH102 cells we isolated, where one of the alleles of *Hmga2* has an insertional mutation, the Mlh1-dependent phosphorylation of H2AX was also found to be decreased compared with the parental cell line, YT102 (Supplementary Fig. S2). These results imply that the HMGA family proteins function prior to ATR/CHK1 activation in the DNA damage signaling at an early step of O^6^-meG-induced apoptosis.

### Effects of apoptosis-related events in *HMGA*-knockdown cells

**Figure 5:**
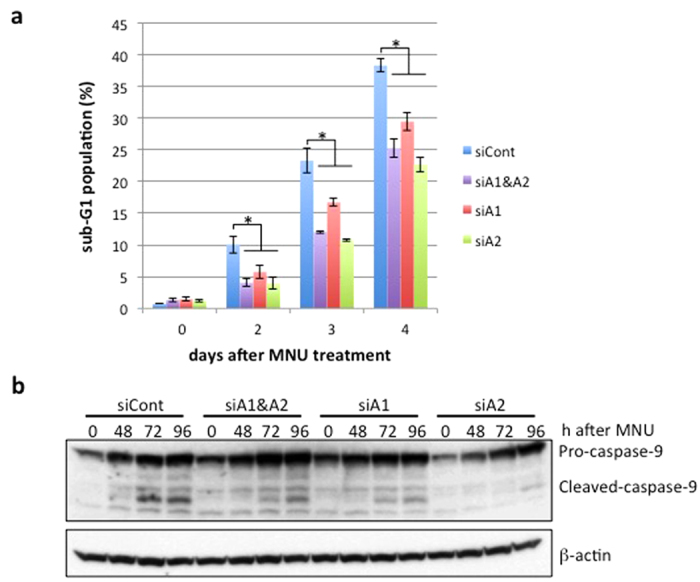
The suppression of apoptosis-related activities in *HMGA*-knockdown cells. (**a**) The sub-G_1_ population in *HMGA*-knockdown cells after MNU treatment. The control and *HMGA*-knockdown cells were treated with 0.4 mM MNU for 1 h and incubated for 0, 2, 3, and 4 days. The cells were harvested and subjected to flow cytometry. The mean values of the sub-G_1_ population obtained from three independent experiments and the standard deviation are shown. Blue bars, negative control; purple bars, *HMGA1-* and *HMGA2*-double knockdown; red bars, *HMGA1*-knockdown; green bars, *HMGA2*-knockdown. Significance value of *P < 0.05 relative to siCont. (**b**) The activation of caspase-9 after MNU treatment. The control and *HMGA*-knockdown cells were treated with 0.4 mM MNU for 1 h and collected at the indicated times after the treatment. Whole-cell extracts were prepared and subjected to SDS-PAGE followed by immunoblotting with anti-caspase-9 antibody. β-actin was the loading control.

We further examined the effects of *HMGA*-knockdown on the appearance of a sub-G_1_ population and the activation of caspase-9, both of which are hallmarks for the induction of apoptosis. A flow cytometric analysis showed that the sub-G_1_ population increased gradually after MNU treatment in cells transfected with either type of siRNA (Fig. 5a). However, the degree of increase was significantly slighter in the *HMGA*-knockdown cells than in the control cells. In the *HMGA1/HMGA2*-double knockdown cells, the degree of reduction (12.0%) at Day 3 after MNU treatment was almost half that of control (23.3%). Similar levels of reduction were obtained in cells introduced with siHMGA1 (16.7%) and siHMGA2 (10.7%).

We next analyzed the activation of caspase-9 in *HMGA*-knockdown cells. An immunoblotting analysis using an antibody that recognizes both pro- and cleaved-caspase-9 revealed that the cleavage of caspase-9 started at 48 h and became more evident at 72 and 96 h after MNU treatment in siCont-transfected cells (Fig. 5b). In contrast, in all types of *HMGA*-knockdown cells, the signals for cleaved-caspase-9 at 72 and 96 h were significantly weaker than in the controls. Furthermore, mitochondrial outer membrane permeabilization following MNU treatment was also suppressed in *HMGA1/HMGA2*-knockdown cells compared to the control (Supplementary Fig. S3). These results are consistent with the above-mentioned view that HMGA family proteins may function at an early step of apoptosis.

## Discussion

In the present study, the gene-trap mutagenesis screening of mouse-derived cells revealed *Hmga2* as a new gene functioning in the MNU-induced apoptotic pathway. An insertional mutation in the *Hmga2* gene rendered cells significantly resistant to MNU, with concomitant reduction in the activation of caspase-3 even after MNU treatment. We further showed that HMGA1, another HMGA family protein, also functions in the induction of apoptosis in response to O^6^-meG. HeLa MR cells transfected with siRNA specific for either *HMGA1* or *HMGA2* acquired certain degrees of resistance to MNU, and double-knockdown cells treated with both siHMGA1 and siHMGA2 exhibited further resistance to the agent, implying that these two proteins cooperate in the process of apoptosis triggered by MNU.

**Figure 6:**
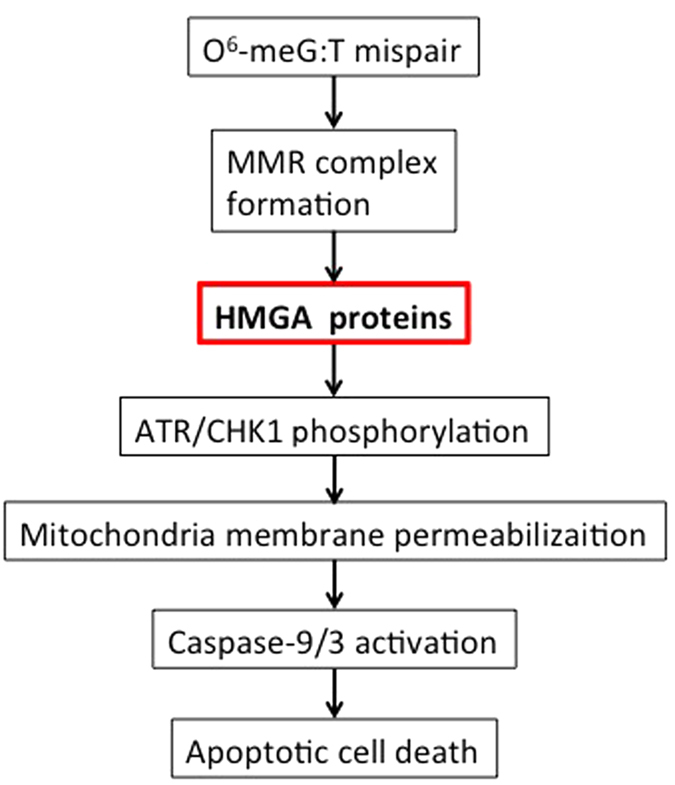
A model for the apoptosis pathway induced by DNA base mismatch. One possible role of HMGA proteins is presented.

To obtain further evidence supporting the notion that HMGA proteins are involved in the O^6^-meG-induced apoptosis, we examined several apoptosis-related events. Our results showed that not only the phosphorylation of ATR, CHK1, and H2AX but also the appearance of the sub-G_1_ population and activation of caspase-9 occur in HMGA-dependent manners. In contrast, the formation of the MMR complex takes place normally in HMGA-suppressed cells. Taken together, these findings suggest that, in the pathway of apoptosis induction, HMGA proteins function at a step immediately after the MMR complex formation and prior to the phosphorylation of ATR/CHK1 as shown in a model for the transfer of an apoptosis induction signal (Fig. 6). The ATM/CHK2 axis is also activated at a later time point than that of ATR/CHK1 in the course of O^6^-meG-induced apoptosis^26^. It is not still clear whether HMGA proteins are involved in the activation of the ATM/CHK2 signaling pathway during apoptosis triggered by O^6^-meG.

HMGA proteins are abundant non-histone chromatin proteins that contain three DNA binding domains, named AT-hooks, and a highly acidic carboxy-terminal region. Although HMGA proteins do not possess intrinsic transcriptional regulatory activity, these proteins have been reported to interact with transcription factors and regulate gene transcription^19^,^20^. It has also been shown that HMGA proteins bound to chromatin recruit chromatin remodeling factors and histone chaperons, thereby enhancing the alteration of chromatin structure^27^,^28^. It is uncertain, at present, how these activities of HMGA proteins are related to the function of the proteins in the induction of apoptosis. Some other activities of HMGA proteins may also be involved in the activation of DNA damage signaling.

HMGA proteins are implicated in predisposition to tumors^29^. Neoplastic phenotypes appeared in *Hmga1*^−/−^ and *Hmga1*^+/−^ mice, suggesting a possible role of HMGA proteins in tumor suppression^30^. The rearrangement of the *HMGA2* sequence, which yields a truncated protein lacking the C-terminus tail, has been found in benign tumors^31^. It has also been reported that the overproduction of HMGA proteins correlates with the metastatic ability of malignant tumors^32^,^33^,^34^. Notably, the mouse KH102 cells isolated as an MNU-resistant clone in the present gene-trap screening had an insertion of vector DNA in the third intron of one of the *Hmga2* alleles, thereby yielding a truncated form of the protein (see Fig. 1b). The truncated protein might compete with the full-length form of Hmga2 for DNA binding and inhibit activities of the full-length one. The dramatic suppression in the induction of apoptosis exhibited by KH102 cells might be related to the predisposition to the development of human benign tumors, in which a chimeric or a truncated form of HMGA2 protein is expressed.

Understanding the precise roles of HMGA family proteins in the process of apoptosis will require learning if any other components are recruited to damaged chromatin through the interaction with HMGA proteins. Studies along this line are in progress in the laboratory.

## Methods

### Cell lines and cell culture

YT102 and YT103 were established from mouse fibroblasts derived from the lung tissue of *Mgmt*^−/−^ and *Mgmt*^−/−^ *Mlh1*^−/−^ mice, respectively^35^. A human-derived HeLa MR cell line defective in the MGMT function was obtained from H. Hayakawa^36^. All of the cell lines were used from our laboratory stocks and cultivated at 37 °C in 5% CO_2_ in Dulbecco’s modified Eagle’s medium (Wako) supplemented with 10% fetal bovine serum and 1% penicillin-streptomycin (Thermo Fisher Scientific).

### Gene-trap mutagenesis

Gene-trap mutagenesis was performed as described previously^12^. Briefly, the retrovirus vector pLHΔU3L carrying a promoter-less hygromycin B-resistance gene was introduced into ΨMP34 retrovirus-packaging cells (Takara Bio Inc.), and retroviral particles were produced. YT102 cells defective in *Mgmt* were infected with the retrovirus and incubated for 15 h. The cells were selected in a medium containing 0.6 mg/ml of hygromycin B for 55 h and then treated with 0.4 mM MNU in serum-free medium for 1 h, followed by further incubation in drug-free medium. The colonies formed were subjected to a survival assay with various concentrations of MNU, and MNU-resistant clones were obtained.

### Survival assay

The cells were treated with various concentrations of MNU in serum-free medium containing 0.02 M HEPES-NaOH (pH 6.0) for 1 h at 37 °C and then cultivated in a complete medium for 10 days at 37 °C. The number of colonies formed was counted, and the survival rates were calculated.

### siRNA transfection

Silencer siRNA for *HMGA1* gene, 5′-CCUGGGAUCUGAGUACAUATT-3′, and Stealth RNAi for the *HMGA2* gene, 5′-GGCCAAGAGGCAGACCUAGGAAAUG-3′, were purchased from Thermo Fisher Scientific. After culturing 8 × 10^4^ cells per well in a 24-well plate for 1 day, the cells were transfected with 40 nM siRNA using the Lipofectamine RNAiMAX reagent (Thermo Fisher Scientific) in accordance with the manufacturer’s protocol. For the control transfection, Silencer select negative control No. 1 siRNA (Thermo Fisher Scientific) was used.

### Immunoblotting

Whole-cell extracts were prepared on culture dishes by adding 2 × SDS sample buffer containing 100 mM Tris-HCl (pH 6.8), 2% SDS, 20% glycerol, 2% β-mercaptoethanol, and 0.4 mg/ml bromophenol blue, followed by boiling for 10 min. The extracts were subjected to SDS-PAGE, and the proteins were transferred onto a PVDF membrane (BioRad). Anti-MSH2 (Thermo Fisher Scientific), anti-MSH6, anti-PMS2, anti-MLH1 (BD Biosciences), anti-HMGA1, anti-HMGA2, anti-phospho-S317-CHK1, anti-CHK1, anti-caspase-9, anti-H2AX (Cell Signaling), anti-FLAG M2 (Sigma), anti-phospho-T1989-ATR (GeneTex), anti-γH2AX (Merck Millipore), anti-ATR (Santa Cruz Biotech.) and anti-β-actin (Sigma) were used as primary antibodies.

### Quantitative real-time PCR

Total RNA from siRNA-transfected cells were prepared using an RNeasy mini kit (Qiagen) and used to synthesize cDNA by PrimeScript Reverse Transcriptase (Takara Bio Inc.). SYBR Premix EX Taq II (Takara Bio Inc.) was used for real-time PCR with the 7500 Real Time PCR System (Applied Biosystems). The PCR primers for *HMGA2* were 5′-AAGTTGTTCAGAAGAAGCCTGCTCA-3′ and 5′-AACTGCTGCTGAGGTAGAAATCGAA-3′, and for *GAPDH* were 5′-GCACCGTCAAGGCTGAGAAC-3′ and 5′-ATGGTGGTGAAGACGCCAGT-3′, all purchased from Takara Bio Inc.

### Chromatin immunoprecipitation

Following treatment with control- or *HMGA*-siRNA, HeLa MR cells expressing Flag-tagged PMS2 were exposed to 0.2 mM MNU for 1 h and further incubated in complete medium. The cells were permeabilized on a dish with buffer A (20 mM HEPES–KOH [pH 7.5], 5 mM KCl, 1.5 mM MgCl_2_, and 0.1 mM dithiothreitol) containing 100 μg/ml of digitonin (Wako) and then treated with 1% formaldehyde (Wako) for 5 min at room temperature. After the addition of 1 M Tris-HCl (pH 8.0), the cells were harvested and collected by centrifugation at 3,000 *g* for 5 min at 4 °C. The cell pellet was suspended in buffer B (50 mM Tris-HCl [pH 8.0], 150 mM NaCl, 0.5% sodium deoxycholate, 1% NP40) containing a protease inhibitor cocktail (Roche Diagnostics) and then sonicated. The material was centrifuged at 20,000 *g* for 10 min at 4 °C, and the supernatant fraction was used as the chromatin extract. For immunoprecipitation, anti-FLAG M2-agarose (Sigma) was added to the chromatin extract and incubated for 12 h at 4 °C. After extensive washing of the beads with buffer B, the proteins bound to the beads were eluted in 40 μl of 2 × SDS sample buffer and subjected to immunoblotting.

### Analyses of apoptosis-related events

For the detection of the sub-G_1_ population, the cells were treated with 0.4 mM MNU as described above and incubated in a complete medium for the indicated times. The cells were collected; suspended in PBS containing 0.1% Triton X-100, 0.025 mg/ml propidium iodide, and 0.1 mg/ml RNaseA; and then subjected to flow cytometry using a FACS Calibur (BD Biosciences). An assay for caspase-3 activity was performed in accordance with the instructions included with the EnzChek caspase-3 assay kit #2 (Thermo Fisher Scientific).

### Statistical analyses

All of the *P*-values were generated using the two-tailed Student’s *t*-test.

## Additional Information

**How to cite this article**: Fujikane, R. *et al*. Function of high-mobility group A proteins in the DNA damage signaling for the induction of apoptosis. *Sci. Rep.* **6**, 31714; doi: 10.1038/srep31714 (2016).

## Supplementary Material

Supplementary Information
