# Supplementary material for: Function of high-mobility group A proteins in the DNA damage signaling for the induction of apoptosis
Source: Sci Rep. 2016 Aug 19;6:31714. doi: 10.1038/srep31714 (PMC4990841; doi:10.1038/srep31714)
Supplement: Supplementary Information [file srep31714-s1.pdf]

# Supplementary Information

## Function of high-mobility group A proteins in the DNA damage signaling for the induction of apoptosis

Ryosuke Fujikane[1], Kayoko Komori[3], Mutsuo Sekiguchi[2], and Masumi Hidaka[1,3]*

[1]Department of Physiological Science and Molecular Biology and [2]Advanced Science Research Center, Fukuoka Dental College, Fukuoka, 814-0193, Japan, [3]Department of Molecular Biology, Biomolecular Engineering Research Institute, Suita, 565-0874, Japan

*Corresponding should be addressed to M.H. (email: hidaka@college.fdcnet.ac.jp)

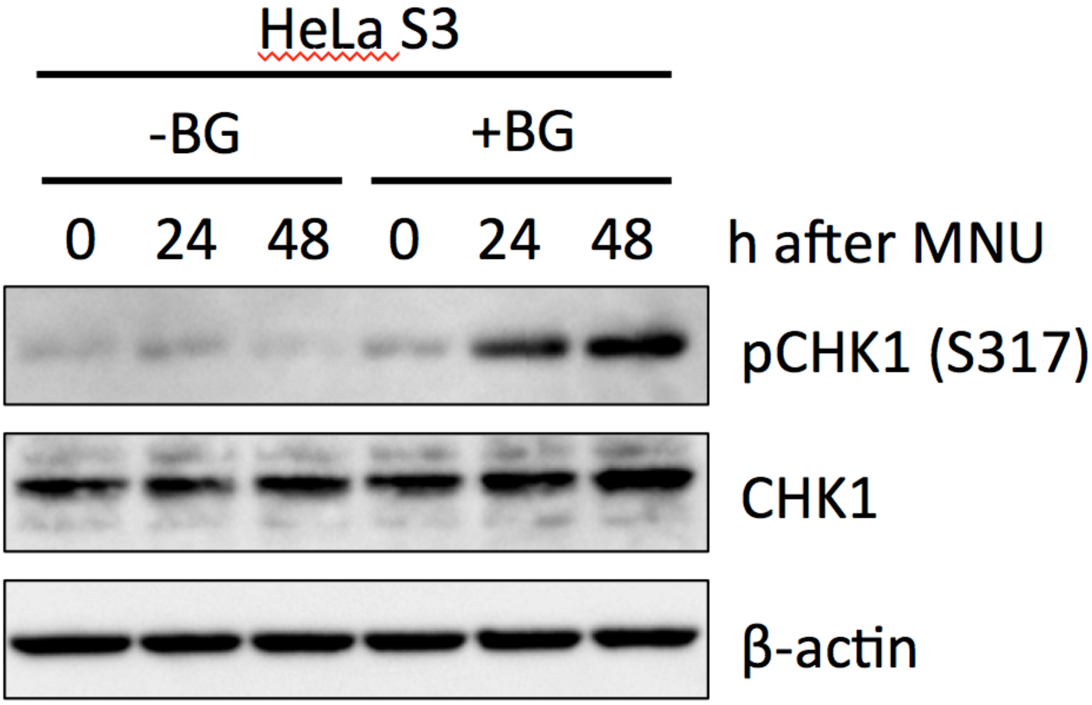


**Supplementary Figure S1. O⁶-methylguanine-dependent activation of CHK1.** MGMT-proficient HeLa S3 cells were treated with or without 25 μM of $O^6$-benzylguanine (Sigma) for 2 h, and then treated with 0.2 mM MNU in serum-free medium containing 0.02 M HEPES-NaOH (pH 6.0) for 1 h at 37 °C. After cultivation in a complete media in the presence or absence of 25 μM of $O^6$-benzylguanine for 24 or 48 h, whole-cell extracts were prepared by lysing the cells by adding 2 × SDS sample buffer containing 100 mM Tris-HCl (pH 6.8), 2% SDS, 20% glycerol, 2% β-mercaptoethanol, and 0.4 mg/ml bromophenol blue, followed by boiling for 10 min. The whole-cell extracts were subjected to SDS-PAGE, and immunoblotting was performed using antibodies specific for phospho-serine-317-CHK1 (Cell Signaling) and total CHK1 (Santa Cruz Biotech.) proteins. β-actin (Sigma) was the loading control.

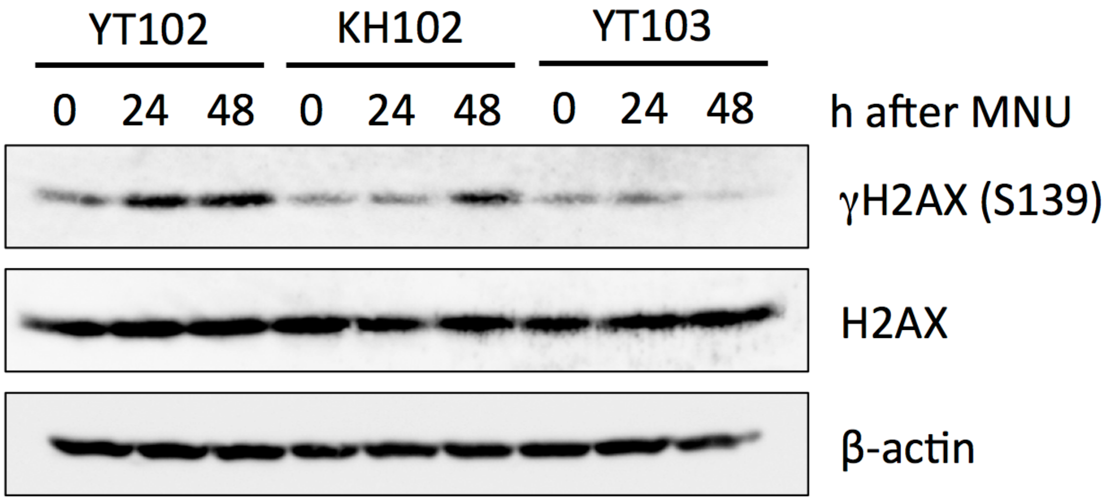


**Supplementary Figure S2. The DNA-damage response in *Hmga2*-deficient cell line KH102.** Mouse fibroblast-derived cell lines YT102 (*Mgmt*$^{-/-}$), KH102 (*Mgmt*$^{-/-}$ *HmgA2*$^{+/-}$), and YT103 (*Mgmt*$^{-/-}$ *Mlh1*$^{-/-}$) were treated with 0.2 mM MNU for 1 h and further cultivated in a complete media for 24 and 48 h. The whole-cell extracts were prepared as described in Supplementary Figure S1. Immunoblotting was performed using antibodies specific for γH2AX (Cell Signaling) and H2AX (Cell Signaling) proteins. β-actin (Sigma) was the loading control.

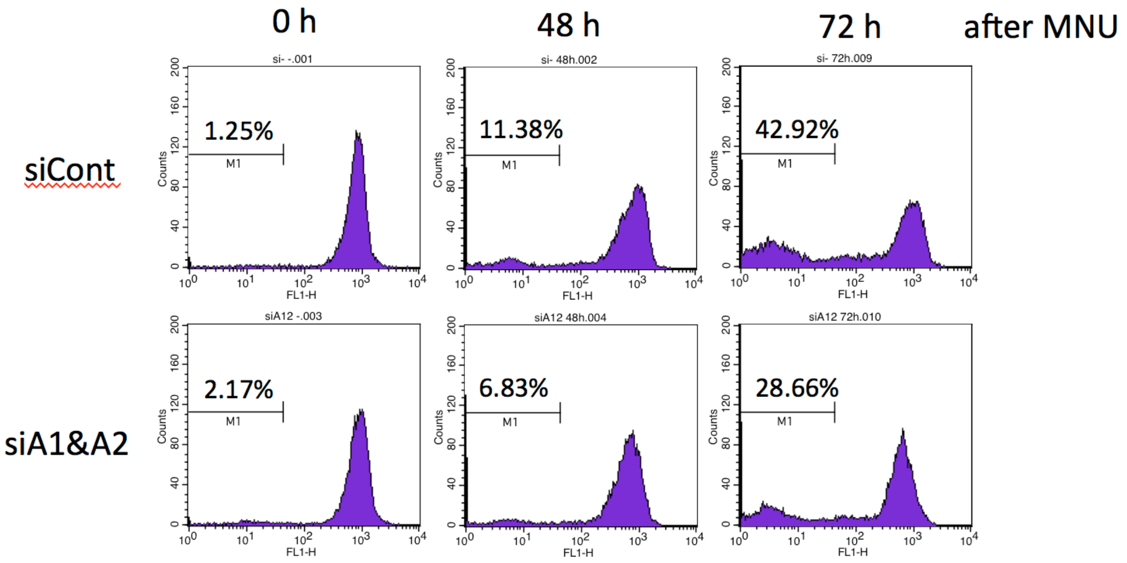


**Supplementary Figure S3. The effects of *HMGA*-knockdown on the mitochondrial outer membrane permeabilization.** siHMGA1/A2-transfected HeLa MR cells were treated with 0.2 mM MNU in serum-free medium containing 0.02 M HEPES-NaOH (pH 6.0) for 1 h at 37 °C, and further cultivated in a complete media for 24 and 48 h. The cells were trypsinized and suspended in 1 ml of PBS. The cells were stained with the membrane-potential-sensitive cyanine dye, $DiOC_2(3)$ in accordance with the instructions (Mitoprobe $DiOC_2(3)$ Assay kit, Thermo Fischer Scientific) and then subjected to flow cytometric analyses. The percentage of mitochondria outer membrane permeabilized cells (M1) were indicated.
